# Self-disseminating vaccines for emerging infectious diseases

**DOI:** 10.1586/14760584.2016.1106942

**Published:** 2015-11-02

**Authors:** Aisling A. Murphy, Alec J. Redwood, Michael A. Jarvis

**Affiliations:** ^a^ School of Biomedical and Healthcare Sciences, Plymouth University, Plymouth, UK; ^b^ The Institute for Immunology and Infectious Diseases, Murdoch University, Murdoch, Western Australia, Australia

**Keywords:** Disseminating, Ebola, emerging infectious disease, zoonosis, cytomegalovirus, wildlife, epidemic, transmission, vaccine, CMV

## Abstract

Modern human activity fueled by economic development is profoundly altering our relationship with microorganisms. This altered interaction with microbes is believed to be the major driving force behind the increased rate of emerging infectious diseases from animals. The spate of recent infectious disease outbreaks, including Ebola virus disease and Middle East respiratory syndrome, emphasize the need for development of new innovative tools to manage these emerging diseases. Disseminating vaccines are one such novel approach to potentially interrupt animal to human (zoonotic) transmission of these pathogens.

Modern human activity is profoundly and irreversibly changing our natural world.[1] In addition to the more publicized global warming and mass extinctions, human activity is altering our relationship with microorganisms to far-reaching effect.[2,3] The impact of this altered relationship can be seen by the startling rise in the rate of emerging infectious diseases (EIDs).[4] Increased global travel, agricultural expansion into wildlife habitats, deforestation and urbanization are all driving humans into greater and more intimate contact with animal populations, creating greater opportunity for human exposure to new microbes.[3–6] Although the particular animal species involved may never be known,[7] the 2014/2015 Ebola virus epidemic in West Africa shows that the global community remains ill-prepared for EIDs.[8,9] This lack of preparedness is multifactorial, but we suggest it includes the current risk adverse nature of scientific funding away from innovative and novel, ‘high-risk, high-reward’ science (i.e., science with a high risk of failure, but which is potentially transformative),[10] as well as insufficient allocation of resources toward the poorer countries that represent the most likely sites of future EIDs of global significance.[4] In the words of Albert Einstein quoted in the recent Advancing Research In Science and Engineering report that highlighted the worrying shift towards ‘low-risk’ science [10]:

If at first the idea is not absurd, then there is no hope for it.

Microbial movement into humans from animals (zoonotic transmission) has always been responsible for the majority of EIDs, including at one time EIDs that form such present day matrix diseases as malaria falciparum,[11] measles and TB.[12] For modern EIDs, it is estimated that over 70% of zoonotic pathogens originate in wildlife, entering either directly from wildlife reservoirs, or indirectly via an intermediate domestic animal host.[4,13] HIV, avian influenza, Hendra and Nipah viruses, SARS and MERS, and Ebola and Marburg filoviruses are all examples of zoonoses currently emerging from wildlife. All of these EIDs present a serious and increasing threat to health, biosecurity and economies worldwide. It is a sobering observation that most modern EIDs were entirely unknown before their entry into the human population. This pattern of emergence of new pathogens of global significance into humans from wildlife is expected to continue.

The task of identifying and preventing zoonoses is daunting, particularly given that the emerging pathogen may be completely unknown. The availability of limited resources and allocation of these resources over short-term funding periods further compounds the problem.[8,9] Recently, a landmark study from Daszak and colleagues examining conditions associated with emergence of >300 EIDs over the past 60 years highlighted the ineffectiveness with which resources for control of EIDs are being allocated.[4] Allowing for reporting bias, this study indicated that emergence of pathogens from wildlife, which represents the greatest threat to global health, is not uniform, but instead is localized to distinct geographic ‘hotspots’ in Africa, Asia and South America. These ‘high-risk’ EIDs were also shown to be biased toward a few ‘high-value’ wildlife species (bats, rodents and non-human primates (NHPs)). This realization that global resources need to be allocated toward countries that have the highest likelihood of high-risk EIDs [4] has led to the recent implementation of surveillance programs (e.g., USAID Emerging Pandemic Threats (EPT) program (superseded by EPT-2 in 2014)) targeting ‘high-value’ target wildlife species in ‘hotspot’ areas prone to EIDs. However, the recent 2014/2015 Ebola virus epidemic emphasizes the need for further investment in such EID surveillance and control programs.

Surveillance is only one component of a successful control program. Following identification, an emerging pathogen must then be controlled. For wildlife pathogens emerging through intermediate domestic animal host species, such as Nipah virus in swine or avian influenza in poultry, effective containment has been achieved either by mass vaccination using conventional vaccines, or by large-scale culling.[5,14] Although costly, the demonstrated ability to successfully contain these EIDs at source suggests the utility of these procedures for EID control in some domestic animals.[5] In contrast, restricted accessibility and idiosyncrasies of natural habitats present unique problems to the control of pathogens within wildlife species. For example, large-scale oral vaccination using vaccine-laden bait has been highly successful in eliminating rabies in wild fox populations in western Europe (for review, see [15]). Similarly, culling of European badgers to control the epizootic spread of *Mycobacterium bovis* to cattle has had some success in the control of bovine TB in Ireland and regions of the UK (for review, see [16]). However, the effectiveness of badger culling is variable and somewhat counterintuitively has also been associated with increased, rather than decreased, bovine TB incidence in some regions.[16] This unanticipated effect is believed to be due to the increased movement of surviving badgers following disruption of their social groups (perturbation effect) [16] – with levels of perturbation varying depending on landscape and intensity of culling. Culling of foxes to combat rabies prior to implementation of mass vaccination similarly led to an increased incidence of rabies in foxes.[15]

In non-temperate ‘hotspot’ regions of Africa, Asia and South America, the ability to contain even known EIDs such as Ebola virus in wildlife is currently not possible. Management of diseases that involve livestock in these regions, such as Rift Valley fever and Crimean Congo hemorrhagic fever, pose similar problems,[17,18] in that conventional vaccines are not suited for use in these environments. A major limitation of conventional vaccination is the requirement for individual inoculation of each animal (directly or via bait) for induction of immunity, which is costly and/or impractical for the target species most frequently involved in high-risk EIDs.[4] Combined with the anticipated intense competition for vaccine bait by non-target species, vaccine temperature lability issues [19] may also undermine the effectiveness of baiting strategies in hotspot regions – especially for far-ranging, low population density animals such as NHPs, which may take several days to come across the vaccine bait.

## Self-disseminating vaccine vectors

Even with programs such as EPT, prediction of which animal pathogens will become established as globally significant EIDs within the human population still remains beyond our capability. However, pathogens emerging from an animal source are often initially poorly adapted to their new human host in terms of sustained human to human transmission.[20] Mechanisms involved in adaptation are unclear and will presumably be idiosyncratic to the particular emerging pathogen, but have been suggested to impart a requirement for repeated introductions into the human population before a successful adaptation event results in full human adaptation.[20] This requirement may provide a potential ‘window of opportunity’ for immunological targeting of the pathogen within the animal transmission species, thereby stemming its continued zoonotic flow prior to acquisition of full adaptation to humans. Self-disseminating vaccines are a vaccine strategy that may in some instances be better suited than conventional vaccines to immunologically contain emerging pathogens within their non-human host in challenging under-resourced ‘hotspots’. Disseminating vaccines are designed to exploit the ability of replicating virus-based vectors to spread through their animal host populations without the need for direct inoculation of every animal. In this strategy, vaccination of a limited number of ‘founder’ animals is used for initial introduction of the vaccine into the target population. As the vaccine is engineered to express target antigens from the EID pathogen of interest, its spread from vaccinated to non-vaccinated animals will result in coordinated spread of EID-specific immunity throughout the targeted animal population.

### Myxoma virus-based vaccines for myxomatosis and rabbit hemorrhagic disease virus

The earliest disseminating vaccine for animals was designed to target two highly lethal rabbit-specific EIDs in the European rabbit population, myxoma virus (MV) and rabbit hemorrhagic disease virus (RHDV).[21] The vaccine was based on a naturally attenuated MV strain (strain 6918) selected for low virulence (non-lethality), high immunogenicity, and maintenance of horizontal transmission.[22] MV6918 is essentially identical to the highly pathogenic wild-type strain except for disruption of four genes, two of which are known virulence factors.[23] MV6918 was able to protect against lethal MV challenge following vaccination using direct inoculation. Importantly, MV6918 was transmitted to >50% of co-housed rabbits (assessed by sero-conversion), and immunity conferred by transmission was protective.[22] Onward transmission was less efficient (approx. 12%) and was no longer protective. MV1698 was subsequently engineered to express RHDV capsid protein as a transmissible bi-valent vaccine against both RHD and myxomatosis.[21,22] Under laboratory conditions, the MV6918VP60-T2 bivalent vaccine was shown to exhibit similar characteristics to MV6918. Direct inoculation was immunogenic and protective in essentially all animals with >50% transmission from directly inoculated to co-housed rabbits and a substantial drop in onward transmission.[21] MV6918VP60-T2 was shown to perform in a remarkably comparable fashion in a limited field trial performed on an island with an estimated population of 300 wild European rabbits with the vaccine showing maintained avirulence, high immunogenicity following direct inoculation and >50% transmission rate.[24]

In the above studies, MV was selected as the genetic basis for the disseminating vaccine due its ability to spread through rabbit populations. High species specificity of MV for rabbits also decreased the potential for spread to ‘off-species’ targets within the environment. However, use of a normally virulent pathogen for the host species being targeted as the self-disseminating vaccine platform necessarily required use of an attenuated MV strain. This requirement had a clear impact on the disseminating capacity of the MV6918-based vaccine. More recent self-disseminating vaccine approaches have used cytomegalovirus (CMV), which is a beta-herpesvirus, as the disseminating vaccine platform. Similar to MV, CMVs are immunogenic and spread efficiently through their host species.[25–27] However, CMV infection is normally benign in the healthy host. This important difference removes the need to use attenuated strains, thereby potentially enabling use of wild-type CMVs with preserved animal-to-animal transmission characteristics. Similar to MV, CMVs are also highly species-restricted with each mammalian host species studied carrying its own CMV.[25] The species barrier for CMV appears remarkably robust, with direct experimental inoculation being unable to establish off-species infection even between closely related rhesus and cynomolgous macaque CMVs (90% identical at the nucleotide level).[28] A recent study showed this strict species restriction to extend to CMVs in the wild, with the absence of cross-species CMV transmission even between chimpanzees and monkey prey species involved in an intimate NHP predator–prey relationship in the Tai Forest National Park, Cote d’Ivore.[29] CMVs are also ubiquitous within their host species,[25] which allows vaccines to be engineered from CMV strains already endemic within the target species. This helps remove concerns associated with introduction of new viruses into animal populations with which there is no long established biological relationship.

### Murine cytomegalovirus-based immunocontraceptive vaccine for domestic mouse (Mus domesticus) plagues

Although not targeting an EID, over a decade of work toward the use of a murine CMV (MCMV) as a viral vectored immunocontraceptive vaccine to control mouse plagues in Australia gives some insight into the application of disseminating vaccines to target high risk pathogens for EID control. Mice directly infected with MCMV strains expressing female mouse fertility antigens develop prolonged – essentially life-long – infertility.[30] Immunocontraception was dependent on antibody production and led to the ablation of ovarian follicles.[31,32] Despite the success of MCMV as an injectable vaccine, lack of direct transmission to uninfected mice under laboratory conditions has been a hurdle to its further development. Transgene expression by MCMV is frequently associated with salivary gland attenuation (a major organ involved in animal-to-animal transmission of CMV). However, even low passaged, non-genetically manipulated wild-type strains of MCMV transmit poorly under laboratory conditions.[32] Therefore, it is not clear if poor transmission of vaccine strains is due to genetic manipulation of the virus and/or reflects a general lack of viral transmission under laboratory conditions. It is possible that the inability to transmit under laboratory conditions may be due to transmission characteristics unique to rodent CMVs. Similar to the situation with MCMV in mice,[33] transmission of Sin Nombre hantavirus (SNV) in deer mice could not be demonstrated under standard laboratory conditions. However, efficient transmission was observed following co-housing in outdoor enclosures and correlated with the number of aggressive encounters enumerated by the number of biting wounds.[34]

### Deer mouse CMV-based vaccine to interrupt Sin Nombre hantavirus zoonotic transmission

The first studies using CMV as a disseminating vaccine targeting a human EID used CMV from deer mice (Peromyscus CMV (PCMV)) to target SNV in the wild deer mouse SNV transmission species. Using a PCMV expressing the SNV envelope glycoprotein G1, the PCMV(ΔP33:G1EGFP) vaccine induced G1-specific antibodies following direct inoculation of deer mice.[35] PCMV(ΔP33:G1EGFP)-induced immunity was durable, persisting over a 12-month period,[36] but was associated with a lower level of PCMV-specific antibodies compared to the wild-type PCMV.[35,36] An observed delay in replication *in vitro* combined with the lower anti-PCMV antibody levels suggested a level of attenuation. However, PCMV(ΔP33:G1EGFP) was still able to induce G1-specific immunity in healthy deer mice previously infected with either PCMV(ΔP33:G1EGFP) or wild-type PCMV.[35,36] This ability of CMV to re-infect the CMV seropositive host is a characteristic shared by other CMVs, and is critical for use of this virus as a disseminating vaccine platform due to CMVs being ubiquitous within their mammalian hosts.[37] The ability of PCMV(ΔP33:G1EGFP) to transmit G1-specific immunity in co-housed mice, or to protect against SNV challenge has not been determined. However, these studies further suggest the importance of using a non-attenuated virus-based vaccine platform with wild-type characteristics, which is possible with CMV given its benign nature in the healthy host.

### CMV-based vaccine to interrupt Ebola virus zoonotic transmission

A disseminating CMV-based approach is also being developed toward the control of Ebola virus in wildlife reservoir and transmission species in Africa.[38,39] Approximately 30% of past human Ebola virus outbreaks are known to have resulted from the direct handling of infected ape carcasses,[40] identifying apes as a critical wildlife Ebola virus transmission species. Ebola virus is also regarded as a major threat to the survival of African ape populations in the wild.[41] Consequently, a disseminating CMV-based strategy is being developed as part of an ongoing multidisciplinary effort between human health scientists and the conservationists at the World Wildlife Fund to target Ebola virus infection in African great apes (bonobo, chimpanzee and gorilla) and potentially also fruit bats. Fruit bats (*Rousettus aegyptiacus*) are also a known reservoir of Marburg virus [42]; a disseminating vaccine platform targeted at bat roosts may also therefore be suitable to interrupt transmission of this related filovirus. A recent series of studies have shown that a CMV-based vaccine is able to provide protection against Ebola virus challenge following direct inoculation.[38,39] In these studies, a MCMV vector expressing a CD8 + T cell epitope from nucleoprotein (NP) of Ebola virus fused to a non-essential MCMV protein (MCMV/ZEBOV-NP_CTL_) was shown to induce durable NP-specific immunity (>14 months).[38,39] MCMV/ZEBOV-NP_CTL_ vaccinated mice showed no evidence of Ebola virus disease (EVD) following lethal Ebola virus challenge. Impressively, 5/8 mice completely controlled Ebola virus infection, with no detectable viremia; the remaining 3 mice showed a 2.8 log reduction in viremia relative to non-vaccinated controls. Protection was long-lived as mice vaccinated with a single dose of MCMV/ZEBOV-NP_CTL_ were protected against EVD following lethal challenge 17 weeks post-vaccination – an attractive quality for a disseminating vaccine to be used in wildlife populations. Studies in the NHP Ebola virus challenge model were recently completed (manuscript under review). In this model, the key question of transmissibility of immunity in CMV seropositive animals (which cannot be assessed in the laboratory mouse model (see above)) can now be addressed in an experimental system more translatable to NHPs in the wild.

Substantial evidence supports the ability of primate CMVs, including human CMV (HCMV), to superinfect the seropositive host. A 2008 study examining HCMV seropositive women showed the frequent presence of multiple glycoprotein N (gN) and/or gB variants within HCMV positive urine and blood samples suggesting that most individuals are infected with multiple HCMV strains.[43] A subsequent study monitoring development of HCMV strain-specific antibody responses in a cohort of healthy seropositive women reported 29% of participants developed new strain-specific antibodies with a mean time of 17.8 months (± 10.3 months) indicating that superinfection is a relatively common event.[44] Superinfection of CMV seropositive NHPs has been demonstrated experimentally in the simian immunodeficiency virus (SIV):rhesus macaque AIDS model following direct inoculation of recombinant rhesus CMV (RhCMV) genetically modified to express SIV antigens.[45] Following superinfection, recombinant RhCMVs were able to establish a persistent long-term infection and induced CD4+ and CD8+ T-cell responses against the expressed SIV antigen comparable to those observed in RhCMV seronegative animals.[45] This ability to induce a robust T-cell response against the ‘new’ heterologous antigen encoded by the RhCMV vector in the presence of prior CMV immunity is notable [46] as it suggests that ‘original antigenic sin’ – a phenomenon first observed for influenza A-specific antibodies,[47] and then for virus-specific T-cell responses in the lymphocytic choriomeningitis virus mouse model,[48] whereby the presence of pre-existing immunity blunts the immune response against a new but cross-reactive antigen – may not apply in this situation. However, the effect on more closely antigenically related heterologous target antigens within the context of CMV infection will need to be determined.

The studies performed in the SIV:rhesus macaque model showed that the ability to superinfect was dependent on genes in the US2-11 region of the genome – a region which contains several genes involved in down-modulation of MHC class I antigen presentation.[49] CD8+ T-cell depletion restored the ability of RhCMVs deleted for US2-11 to superinfect seropositive animals indicating that superinfection was due to viral subversion of the host CD8+ T-cell immune response. Interestingly, following recovery of the CD8+ T-cell response in these animals, the US2-11 deleted viruses were able to persist, which indicates that once established, the host CD8+ T-cell response is unable to clear virus infection. Outside of the MCMV mouse model (see above), the ability of recombinant CMVs to spread between animals has not been tested. However, a recent study investigating transmission of RhCMV in co-housed animals showed that non-recombinant, but tissue culture-passaged RhCMV strains maintain an ability to be shed into bodily fluids (saliva and urine) at levels comparable to those of wild-type RhCMV, provided that a region of the genome encoding several genes involved in tropism and immune evasion (called the UL/b’ region) is intact.[50] CMV transmission is generally believed to involve mucosal exposure to such fluids (as well as genital secretions and breast milk).[25] Consistent with their maintained shedding characteristics, the tissue culture-passaged viruses also retained the ability to spread between co-housed RhCMV-seropositive animals. This observation indicates that it is at least possible for laboratory manipulated CMV strains to maintain the ability for wild-type transmission.

Further studies are needed to ensure recombinant CMVs expressing heterologous target antigens can similarly maintain wild-type transmission and target-specific immune responses following transmission. Experience from studies exploring the use of disseminating vaccines targeting other pathogens (see above) are expected to prove invaluable for these studies, especially in regard to the importance of avoiding vaccine attenuation to maintain wild-type characteristics of CMV transmissibility. Where studied, frequencies of CMV infection from natural transmission in animal populations approach 100%. Consistent with epidemiological studies in humans, a major peak of infection occurs at an early host age, with essentially all US primate center rhesus macaques being RhCMV seropositive by the age of one year.[37] Environmental stresses, such as SIV infection of chimpanzees, can result in immune suppression of animals in the wild.[51] It will therefore also be important to ensure that any CMV-based vaccine presents no higher risk in immune-compromised animals than the wild-type CMV strains with which they are already infected.

## Expert commentary

It is becoming clear that the emerging pathogens which represent the greatest risk to global health will most likely be transmitted from a few key animal species in poorer areas of the world. Recent history also tells us that these pathogens will probably have never been seen before. The 2014/2015 Ebola virus outbreak shows the difference healthcare infrastructure can have on human-to-human transmission of EIDs. Until the time when overall healthcare infrastructure in *all* countries has been raised to a level that enables identification and control of high-risk EIDs at source, we as a global community will be fated to reactive responses to EID outbreaks. Innovative strategies are therefore urgently required to identify and then pre-emptively control EIDs in these under-resourced ‘hotspot’ regions. Self-disseminating vaccination is one innovative approach that may potentially overcome the problems associated with use of conventional vaccines for pre-emptive pathogen control in ‘high-value’ animal populations within these challenging environments. Although still in relatively early stages, the nascent field of self-disseminating transmissible vaccines has the potential to solve many current intractable public health and conservation problems that cannot be addressed by conventional vaccines (Table 1).

**Table 1.** A non-exclusive list of current emerging zoonotic diseases amenable to targeting with a self-disseminating vaccine strategy.

Zoonosis	Pathogen	Incidence	Primary transmission/reservoir species	Vector	Vaccine	Protective target antigens	Global Annual Impact
Ebola	Virus	Sporadic	Great apes and bats (?)	None	Yes (E*)	GP	High lethality and capacity for spread
Rabies	Virus	55,000 deaths/year	Dogs and wildlife (bats, foxes, skunk and raccoons)	None	Yes Dogs/Fox	G	$583 million [43]
Pandemic/epidemic influenza A	Virus	Sporadic/annual	Pigs and birds (domestic and wild)	None	Yes Human/Fowl	HA/NA	$71–167 billion (US, epidemic) [42]
MERS	Virus	Sporadic	Bats and dromedary camels	None	Yes (E) Mice [53]	S	Unknown, but potential for rapid spread and significant mortality
Cystic echinococcosis (*Echinococcus granulosus*)	Cestode (dog tapeworm)	Unknown	Dogs and sheep	None	Yes (E) Sheep/Dogs	EG95 EgA31 EgTrp Egms	$1.9 billion (Human-associated) $2.1 billion (Livestock) [44]
Cysticercosis *(Taenia solium)*	Cestode (pig tapeworm)	2.5 million >50,000 deaths/year [47]	Pigs	None	Yes (E) Pigs	TSOL18 TSOL45	50% of late-stage epilepsy attributable to neurocysticercosis in endemic areas [46]
Leptospirosis (*Leptospira*)	Bacteria	500,000 cases/year 5–10% mortality [52]	Multiple including rodents and dogs	None	Yes (E) Hamster	LigA, LigB	Unknown
Lassa fever	Virus	300,000 cases/year 2% mortality [51]	Rodents (*Mastomys natalensis*) [50]	None	Yes (E) NHP	GPC NP	Unknown
Bovine TB *(Mycobacterium bovis)*	Bacteria	Variable, but increasing	Domestic cattle and wildlife (badgers)	None	Yes (human) Efficacy unclear	Unknown	$160 million (Livestock, UK) [45]
Chagas disease *(Trpanosoma cruzi)*	Protozoa	>10 million, 50–200,000 deaths/year	Dogs and wildlife	Triato-mine bugs	No	Unknown	$1.3 billion in lost wages/productivity in Brazil alone [48]
Acute sleeping sickness *(Trypanosoma brucei rhodesiense)*	Protozoa	50–70,000 cases/year–5% of total sleeping sickness disease (40% cattle in Uganda are carriers)	Livestock and wildlife	Tse fly	No	Unknown	$4.75 billion due to impact on herd health in sub-Saharan Africa [49]
Chronic sleeping sickness (*Trypanosoma brucei gambiense*)	Protozoa	95% of total sleeping sickness disease	Livestock and wildlife, but not clearly defined	Tse fly	No	Unknown	Unknown

Data taken from Refs: [54–65]. *E = conventional experimental vaccine.

## Five-year view

Identification of geographic ‘hotspots’ and ‘high-value’ wildlife species for EIDs is beginning to enable more informed decisions over allocation of finite resources to protect global health.[3,4] In recognition of the change in thinking, the USAID EPT programs in collaboration with multiple partners aims to ‘monitor for and increase the local capacity in “geographic hot spots” to identify the emergence of new infectious diseases in high-risk wildlife such as bats, rodents, and non-human primates’.[52] Identification of animal pathogens destined to become significant EIDs within the human population still remains beyond current technical capacity. However, the initial poor adaptation of many emerging zoonotic pathogens may provide a window of opportunity during the initial, stuttering transmission phase into humans enabling the nascent EID to be targeted within its animal host at a stage when it is still amenable to control. Following such pathogen identification, a self-disseminating vaccine platform provides one means to pre-emptively control the emerging pathogen. Adaptation of new technologies such as CRISPR/Cas9 will also greatly increase the ease and speed with which these new vaccine vectors can be constructed following identification of a target pathogen.[53] The next five years has the potential to place us in the position of being able to achieve high vaccine coverage against ‘nascent’ zoonotic pathogens in animal species involved in transmission that are otherwise inaccessible to conventional vaccination. For many ‘high-risk’ zoonotic pathogens, this reduction in zoonotic flow into the human population may decrease the probability of complete adaption to the human host with global significance.

## Financial & competing interests disclosure

MA Jarvis and AA Murphy declare conflicts of interest: patents EP1766096B1, EP2497831B1 and application EP2772265A3 (MA Jarvis); and UK applications No. 1508136.7, No. 1514005.6 and No. 1514034.6 (MA Jarvis and AA Murphy). The authors would like to acknowledge support by the School of Biomedical and Healthcare Sciences, Plymouth University (MA Jarvis and AA Murphy), The Marie Skłodowska-Curie Programme (MA Jarvis) and The Institute for Immunology and Infectious Diseases, Murdoch University (AJ Redwood). The authors have no other relevant affiliations or financial involvement with any organization or entity with a financial interest in or financial conflict with the subject matter or materials discussed in the manuscript apart from those disclosed.

**Key issues**

- Emerging infectious diseases (EIDs) place an increasing burden on human health and infrastructure. The increasing rate of EID incidence can be directly linked to human activity.
- Current funding paradigms often focus on wealthy countries rather than developing countries which contain geographic EID ‘hotspots’. This funding paradigm leaves the world ill-prepared to detect and then control these diseases.
- Targeting zoonotic pathogens in wildlife populations by vaccination is a potentially powerful method for reducing transmission of EIDs. Direct vaccination of these animals may be hampered by a number of obstacles, including: poor infrastructure to support necessary cold chains, inaccessibility of animal species, inhospitable terrain and cost.
- Vaccines capable of self-dissemination may overcome many of the hurdles faced by direct administration of conventional vaccines. Self-disseminating vaccines will need to display species specificity, immunogenicity and normal transmission dynamics. Cytomegalovirus (CMV)-based viral vectors potentially fulfill many of these requirements, including being native to and endemic in target animal species.
- ‘Proof of concept’ studies using direct inoculation of CMV-based vaccine vectors have been performed for viral pathogens such as Sin Nombre hantavirus and Ebola virus. CMV-based vaccine vectors have also been used for successful ‘proof of concept’ of viral vectored immunocontraception.
- Production of CMV-based vaccines with proven capacity to transmit is the next step in the development of self-disseminating vaccines.

## References

[CIT0001] **Reference annotations**.

[CIT0002] *** Of interest**.

[CIT0003] **** Of considerable interest**.

[CIT0004] Ceballos G, Ehrlich PR, Barnosky AD (2015). Accelerated modern human-induced species losses: entering the sixth mass extinction. Sci Adv.

[CIT0005] Morens DM, Folkers GK, Fauci AS. (2004). The challenge of emerging and re-emerging infectious diseases. Nature.

[CIT0006] Keesing F, Belden LK, Daszak P (2010). Impacts of biodiversity on the emergence and transmission of infectious diseases. Nature.

[CIT0007] Jones KE, Patel NG, Levy MA (2008). Global trends in emerging infectious diseases. Nature.

